# Deficiency in hormone-sensitive lipase accelerates the development of pancreatic cancer in conditional KrasG12D mice

**DOI:** 10.1186/s12885-018-4713-y

**Published:** 2018-08-07

**Authors:** Mu Xu, Hui-Hua Chang, Xiaoman Jung, Aune Moro, Caroline Ei Ne Chou, Jonathan King, O. Joe Hines, James Sinnett-Smith, Enrique Rozengurt, Guido Eibl

**Affiliations:** 1 Departments of Surgery, David Geffen School of Medicine, University of California, Los Angeles, 10833 Le Conte Ave, CHS 72-236, Los Angeles, CA 90095 USA; 2 Departments of Medicine, David Geffen School of Medicine, University of California, Los Angeles, Los Angeles, CA USA; 3 CURE: Digestive Diseases Research Center, University of California at Los Angeles, Los Angeles, USA

**Keywords:** Pancreatic cancer, Hormone sensitive lipase, Adipose tissue inflammation, Pancreatic inflammation, Animal model

## Abstract

**Background:**

Hormone sensitive lipase (HSL) is a neutral lipase that preferentially catalyzes the hydrolysis of diacylglycerol contributing to triacylglycerol breakdown in the adipose tissue. HSL has been implicated to play a role in tumor cachexia, a debilitating syndrome characterized by progressive loss of adipose tissue. Consequently, pharmacological inhibitors of HSL have been proposed for the treatment of cancer-associated cachexia. In the present study we used the conditional KrasG12D (KC) mouse model of pancreatic ductal adenocarcinoma (PDAC) with a deficiency in HSL to determine the impact of HSL suppression on the development of PDAC.

**Methods:**

*KC;Hsl*^*+/+*^ and *KC;Hsl*^*−/−*^ mice were fed standard rodent chow for 20 weeks. At sacrifice, the incidence of PDAC was determined and inflammation in the mesenteric adipose tissue and pancreas was assessed histologically and by immunofluorescence. To determine statistical significance, ANOVA and two-tailed Student’s *t*-tests were performed. To compare PDAC incidence, a two-sided Fisher’s exact test was used.

**Results:**

Compared to *KC;Hsl*^*+/+*^ mice, *KC;Hsl*^*−/−*^ mice gained similar weight and displayed adipose tissue and pancreatic inflammation. In addition, *KC;Hsl*^*−/−*^ mice had reduced levels of plasma insulin and leptin. Importantly, the increased adipose tissue and pancreatic inflammation was associated with a significant increase in PDAC incidence in *KC;Hsl*^*−/−*^ mice.

**Conclusions:**

HSL deficiency is associated with adipose tissue and pancreatic inflammation and accelerates PDAC development in the KC mouse model.

## Background

Hormone sensitive lipase (HSL) is an intracellular, neutral lipase that catalyzes the hydrolysis of triacylglycerol, diacylglycerol, monoacylglycerol, cholesteryl esters, and retinyl esters [1]. Its activity against diacylglycerol is several-fold higher than against triacylglycerol and monoacylglycerol. Adipose triglyceride lipase (ATGL) and HSL are therefore the major enzymes contributing to triacylglycerol breakdown in the adipose tissue [2]. Interestingly, an increase in the level and activity of HSL has been implicated in the pathogenesis of cachexia [3, 4], a debilitating syndrome characterized by progressive loss of adipose tissue via increased lipolysis [2–5]. Consequently, pharmacological inhibitors of HSL have been proposed for the treatment of cancer-associated cachexia [4] and a number of compounds have been synthesized and characterized [6, 7]. However, the impact of HSL suppression on cancer development has not been examined.

Pancreatic ductal adenocarcinoma (PDAC) is an extremely aggressive disease with an overall 5-year survival rate of about 8% [8]. Currently, it is the fourth leading cause of cancer deaths in both men and women [8]. PDAC mortality is projected to increase, and before the year 2030 it is expected to become the second leading cause of cancer-related deaths [9]. Cachexia is a prominent condition in PDAC that severely restricts therapeutic options. Therefore, the development of pharmacological agents that can attenuate or reverse cachexia in the context of PDAC is of clinical importance. Administration of inhibitors of HSL have been proposed in the management of cachexia [4] but the precise effect of chronic HSL suppression on the progression of PDAC has never been examined.

HSL null mice appear phenotypically normal (with the exception of infertility in males due to severe oligo- or azoospermia) and are resistant to diet-induced and genetic obesity [10]. In order to assess the role of HSL in PDAC development, we generated conditional Kras^G12D^ mice with HSL deficiency. We found that KC mice with HSL deficiency, compared to KC mice with functional HSL, had similar weight gain and enhanced adipose tissue (AT) and pancreatic inflammation. Surprisingly, KC mice with HSL deficiency exhibited an increased incidence of PDAC. Our results strongly indicate that HSL deficiency is sufficient to accelerate PDAC development in KC mice and therefore imply that chronic suppression of HSL has an unrecognized tumor promoting effect in the KC model.

## Methods

### Conditional KrasG12D mouse model with HSL deficiency

**Fig. 1:**
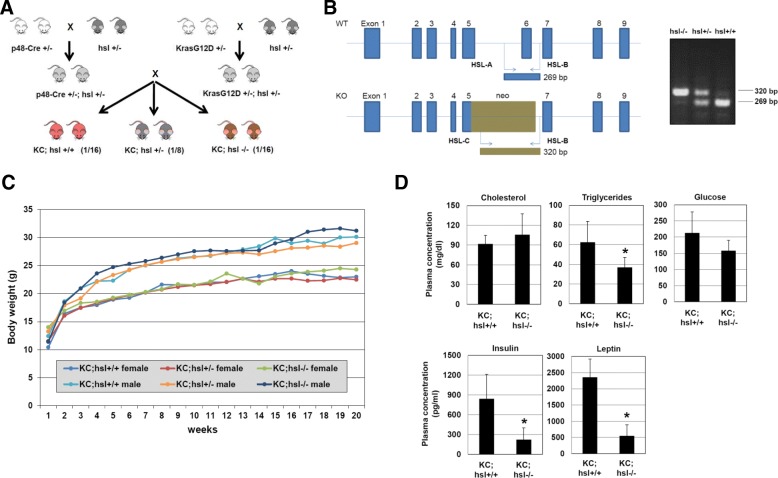
**a** Breeding scheme to generate *KC;Hsl*^*+/+*^, *KC;Hsl*^*+/−*^, and *KC;Hsl*^*−/−*^ mice. **b** Exon structure of wildtype and HSL deficient mice. Replacing portions of exon 5 and the entire exon 6 with a neo cassette renders mice functionally HSL deficient [10]. Representative genotyping result showing a single 320 bp band in *Hsl*^*−/−*^ mice, a single 269 bp band in *Hsl*^*+/+*^ mice, and a double band (320 bp and 269 bp) in *Hsl*^*+/−*^ mice. **c** Weight curves of female and male *KC;Hsl*^*+/+*^ (*n* = 9 males+ 14 females), *KC;Hsl*^*+/−*^ (*n* = 20 males+ 23 females), and *KC;Hsl*^*−/−*^ (*n* = 10 males+ 10 females) mice fed standard rodent chow for 20 weeks. **d** Plasma levels of cholesterol, triglycerides, glucose, insulin, and leptin in *KC;Hsl*^*+/+*^ and *KC;Hsl*^*−/−*^ mice at 6 months (at sacrifice). *N* = 6

The conditional KrasG12D (KC) mouse model from Hingorani and colleagues was used for this study [11]. In the KC (*LSL-KrasG12D;p48-Cre*) strain, expression of oncogenic KrasG12D is activated by Cre-mediated excision of LoxP-Stop-LoxP (LSL) in pancreatic lineages during early embryonic development when the *Ptf1a/p48* promoter is active. HSL deficient mice were kindly provided by Fredric Kraemer at Stanford University [10]. *Hsl*^*+/−*^ mice were crossed into *p48-Cre*^*+/−*^ and *LSL-KrasG12D*^*+/−*^ mice to obtain *p48-Cre*^*+/−*^*;Hsl*^*+/−*^ and *LSL-KrasG12D*^*+/−*^*;Hsl*^*+/−*^ double mutants. The double mutant mice were crossed to generate the desired triple mutant genotypes: *LSL-KrasG12D*^*+/−*^*;p48-Cre*^*+/−*^*;Hsl*^*+/+*^ *(KC;Hsl*^*+/+*^*), LSL-KrasG12D*^*+/−*^*;p48-Cre*^*+/−*^*;Hsl*^*+/−*^ *(KC;Hsl*^*+/−*^*), and LSL-KrasG12D*^*+/−*^*;p48-Cre*^*+/−*^*;Hsl*^*−/−*^ *(KC;Hsl*^*−/−*^*)* (Figs. 1a, b)*.* Mice were fed regular chow beginning at one month of age until 20 weeks of age. We analyzed 23 *KC;Hsl*^*+/+*^, 43 *KC;Hsl*^*+/−*^, and 20 *KC;Hsl*^*−/−*^ mice. Animal studies were approved by the Chancellor’s Animal Research Committee of the University of California, Los Angeles (UCLA) in accordance with the National Institutes of Health Guide for the Care and Use of Laboratory Animals. All mice were sacrificed under general anesthesia with isoflurane. None of the animals died without euthanasia.

### Genotyping analysis

The *LSL-KrasG12D*, *p48-Cre*, and *Hsl* alleles were genotyped by polymerase chain reaction (PCR) analysis as described elsewhere [10, 12].

### Blood metabolic panel

Blood samples were collected from mice by intracardiac puncture at euthanasia. Blood chemistry (plasma cholesterol, glucose, and triglycerides levels) was obtained by the DLAM Pathology & Laboratory Medicine Services at UCLA. Levels of plasma insulin and leptin were determined using the MILLIPLEX MAP Mouse Adipokine Magnetic Bead Panel - Endocrine Multiplex Assay (EMD Millipore, Billerica, MA) based on the manufacturer’s instructions.

### Adipose tissue inflammation

Formalin-fixed AT was paraffin-embedded and sectioned. Hematoxylin and eosin (H.E.)-stained sections were analyzed on a Nikon Eclipse 90i microscope (Nikon, Melville, NY) equipped with NIS AR4.2 software (Nikon). Crown-like structures (CLS) representing inflammatory foci [13, 14] were quantified and described as number per high-power field (hpf). A CLS was defined as one adipocyte surrounded by inflammatory cells at least partially. For each tissue sample, ten images were taken, and the number of CLS per 10 randomly selected adipocytes were quantified.

### Cytokine array

Mesenteric fat homogenates from *KC;Hsl*^*+/+*^ and *KC;Hsl*^*−/−*^ mice were profiled using the Mouse Cytokine Antibody Array, C1000 (RayBiotech, Norcross, GA) following the manufacturer’s instructions. The membrane-based proteomic array detects relative levels of 96 different cytokines and chemokines. The complete list of cytokines and chemokines analyzed can be found here: https://www.raybiotech.com/c-series-mouse-cytokine-array-c1000-2/. Following exposure to horseradish peroxidase (HRP) the membranes were imaged using the ChemiDoc™ Touch Imaging System (Bio-Rad Laboratories, Hercules, CA), and the intensity of signals normalized to the internal positive controls was quantified with Multi Gauge V3.0 software (Fujifilm Life Sciences, Tokyo, Japan).

### Pancreas histology

Formalin-fixed, paraffin-embedded pancreatic tissue sections were stained with hematoxylin and eosin and histologically analyzed in a blinded fashion. Murine pancreatic intraepithelial neoplasia (PanINs) and invasive PDAC were classified according to histopathologic criteria as previously described [15–17].

### Quantification of lipid content

Tissue content of triglycerides (TG), phospholipids (PL), and free fatty acids (FFA) and the fatty acid (FA) profile in each lipid fraction was analyzed by the Vanderbilt Mouse Metabolic Phenotyping Center. Briefly, phospholipids, diglycerides, triglycerides, and cholesteryl esters in the extracted lipids were separated by thin layer chromatography, scraped from the plates and methylated. The methylated FA were then extracted and analyzed on an Agilent 7890A gas chromatograph. FA methyl esters were identified by comparing the retention times to those of known standards. Inclusion of lipid standards with odd chain FAs allowed quantitation of lipids in the sample. Dipentadecanoyl phosphatidylcholine (C15:0), diheptadecanoin (C17:0), trieicosenoin (C20:1), and cholesteryl eicosenoate (C20:1) were the standards used.

### Immunohistochemistry

Paraffin was removed with xylene and graded alcohol. Heat-induced antigen retrieval was performed with citrate buffer, and endogenous peroxidase activity was blocked with 3% hydrogen peroxide. Slides were then incubated overnight with polyclonal rabbit primary antibodies against HSL (Novus Biologicals, Littleton, CO) or monoclonal rabbit primary antibodies against Ki67 (Cell Signaling Technologies, Danvers, MA). Control images were prepared using isotype matched rabbit IgG (Cell Signaling Technologies). Images were analyzed on a Nikon Eclipse 90i microscope equipped with NIS AR4.2 software (Nikon). Ki67 staining was quantified by counting Ki67 positive cells (stromal and epithelial) in 10 high-power fields per tissue section.

### Immunofluorescence

Paraffin was removed with xylene and graded alcohol. Antigen retrieval was performed by using Antigen Unmasking Solution (Vector Laboratories, Burlingame, CA) plus ethylenediaminetetraacetic acid (EDTA). Endogenous peroxidase activity was blocked with 1% hydrogen peroxide. Slides were then incubated overnight with monoclonal rabbit anti-F4/80 antibody (clone SP115, Novus Biologicals) and monoclonal mouse anti-TNF-α (tumor necrosis factor alpha) antibody (clone SPM543, Novus Biologicals). Anti-rabbit IgG antibodies conjugated with Alexa Fluor 555 (Thermo Fisher Scientific, Canoga Park, CA) and anti-mouse IgG antibodies conjugated with Alexa Fluor 488 (Thermo Fisher Scientific) were added at room temperature for one hour. Images were analyzed on a Nikon Eclipse 90i microscope equipped with NIS AR4.2 software (Nikon). F4/80 and TNF-α positive cells were counted in 10 hpf per tissue section and analyzed as 0 (no staining), + (1–5 cells/hpf), ++ (5–10 cells/hpf), and +++ (> 10 cells/hpf).

### Western blot analysis

Mouse tissue samples were homogenized in radio-immunoprecipitation assay (RIPA) buffer containing mixture of protease and phosphatase inhibitors (Roche Applied Science, Basel, Switzerland). Tissue homogenates were resolved by SDS-PAGE, electrophoretically transferred onto nitrocellulose membranes, and then immunoblotted for the proteins of interest using the following primary antibodies: Peroxisome proliferator-activated receptor gamma (PPAR-γ) and p44/42 MAPK from Cell Signaling Technologies, and HSL from Novus Biologicals. After incubation with secondary antibodies, the immune-reactive bands detected with enhanced chemiluminescence reagents were imaged and analyzed by the ChemiDoc™ Touch Imaging System (Bio-Rad Laboratories).

### PCR analysis of *Hsl*

Total RNA from tissue or cell lysates were extracted using RNA purification kits (Biomiga, Inc., San Diego, CA). Reverse transcription was performed with the iScript reverse transcription supermix (Bio-Rad Laboratories). The synthesized cDNA was used as template for the PCR analysis of *Hsl* gene expression. The iTaq™ Supermix (Bio-Rad Laboratories) was used for amplifications. All reactions were performed on the Bio-Rad iQ™5 system. Primers for mouse *Hsl*: forward, 5′- GCAGTGGTGTGTAACTAGGATTG-3′, and reverse, 5′- CGCTGAGGCTTTGATCTTGC -3′ (spanning exons 1 and 2).

### Statistical analysis

Data are presented as mean ± SD. Statistical significance was determined by one-way (or two-way) ANOVA and two-tailed Student’s *t*-tests assuming unequal variances. For the comparison of PDAC incidence, a two-sided Fisher’s exact test was performed. Significance (*p-*value less than 0.05) was indicated with an asterisk (*).

## Results

### Weight gain and metabolic parameters in KC mice with HSL deficiency

After weaning female and male *KC;Hsl*^*+/+*^, *KC;Hsl*^*+/−*^, and *KC;Hsl*^*−/−*^ mice were fed regular rodent chow for 20 weeks. Although females gained less weight than male mice, *KC;Hsl*^*+/+*^, *KC;Hsl*^*+/−*^, and *KC;Hsl*^*−/−*^ mice within each gender group gained weight similarly throughout the study period (Fig. 1c), which is in accordance with previous reports [10, 18]. Compared to *KC;Hsl*^*+/+*^ mice, *KC;Hsl*^*−/−*^ mice showed reduced plasma TG levels, while cholesterol levels were unchanged (Fig. 1d). Our studies demonstrated reduced plasma insulin levels in *KC;Hsl*^*−/−*^ mice with normal glucose concentrations (Fig. 1d), suggesting enhanced insulin sensitivity. Furthermore, *KC;Hsl*^*−/−*^ mice had reduced plasma levels of leptin (Fig. 1d), a hormone secreted primarily by adipocytes of white adipose tissue (WAT).

### KC mice with HSL deficiency display enhanced AT inflammation

**Fig. 2:**
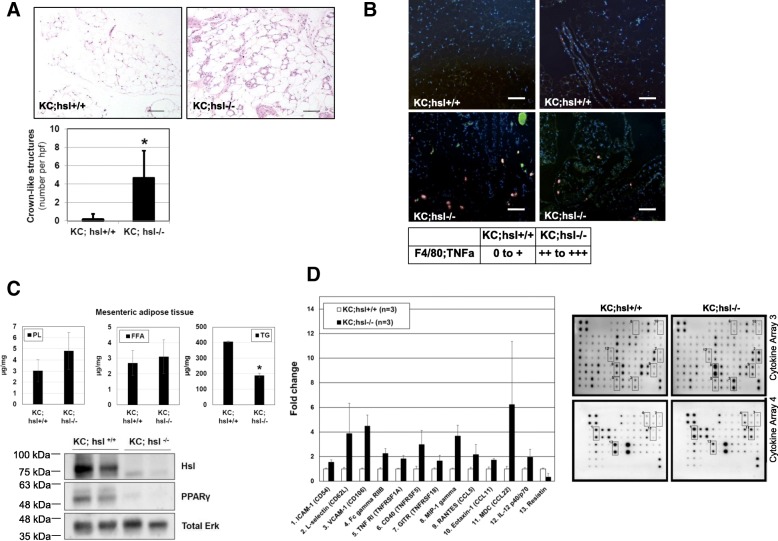
**a** Representative histology (H.E. staining) of the mesenteric WAT of *KC;Hsl*^*+/+*^ and *KC;Hsl*^*−/−*^ mice. Scale bar represents 100 μm. Quantification of crown-like structures (CLS) in *KC;Hsl*^*+/+*^ and *KC;Hsl*^*−/−*^ mice (right). *: *p* < 0.01. **b** Immunofluorescence staining of mesenteric WAT of *KC;Hsl*^*+/+*^ and *KC;Hsl*^*−/−*^ mice (two representative mice in each group). Red and green staining denote F4/80 (macrophage marker) and TNF-α (as a marker of tissue inflammation), respectively. Scale bar represents 100 μm. Semi-quantitative analysis of TNF-α expressing macrophages (orange staining) in *KC;Hsl*^*+/+*^ and *KC;Hsl*^*−/−*^ mice (table below). **c** Quantification of PL, TG, and FFA content in mesenteric WAT of *KC;Hsl*^*+/+*^ and *KC;Hsl*^*−/−*^ mice (*n* = 5). Western blot analysis of HSL and PPAR-γ (total ERK as loading control) in *KC;Hsl*^*+/+*^ and *KC;Hsl*^*−/−*^ mice (lower left). **d** Changes (≥ 50% increase or decrease compared to *KC;Hsl*^*+/+*^) in several cytokines and chemokines in the mesenteric WAT of *KC;Hsl*^*−/−*^ mice as detected by a Mouse Cytokine Antibody Array (*n* = 3 biological replicates). Representative array blots (Cytokine Array 3 and 4) in *KC;Hsl*^*+/+*^ and *KC;Hsl*^*−/−*^ mice (right)

We have previously reported an enhanced depot-specific AT inflammation during diet-induced obesity (DIO) in KC mice [14]. In the present study, histological examination revealed increased WAT (mesenteric depot) inflammation in *KC;Hsl*^*−/−*^ mice as assessed by quantification of CLS (Fig. 2a). The increase in CLS was accompanied by an enhanced presence of TNF-α expressing F4/80-positive cells (macrophages) in the mesenteric WAT of *KC;Hsl*^*−/−*^ mice as assessed by immunofluorescence (Fig. 2b). Detailed tissue lipid analysis revealed decreased TG content in mesenteric WAT of *KC;Hsl*^*−/−*^ mice as well as reduced expression of pro-adipogenic PPAR-γ (Fig. 2c), confirming the impairment of adipogenesis in HSL null mice [18]. The anti-adipogenic phenotype associated with HSL deficiency is consistent with the role of HSL in providing intrinsic ligands for PPARγ through release of FAs [19]. The PL content in mesenteric WAT was unchanged in *KC;Hsl*^*−/−*^ mice, reflecting the lack of phospholipase activity of HSL. Despite the physiological role of HSL in FA release, the levels of FFA in the mesenteric WAT of *KC;Hsl*^*−/−*^ mice were unaltered (Fig. 2c). Due to limited amount of tissue available for each analysis, we did not measure the intracellular levels of diacylglycerols, which are known to accumulate in tissues of *Hsl*^*−/−*^ mice due to the importance and preference of HSL to hydrolyze diacylglycerols [20]. Using a Mouse Cytokine Antibody Array, multiple changes in cytokines and chemokines were detected in the mesenteric WAT of *KC;Hsl*^*−/−*^ mice (Fig. 2d). Significant elevations of adhesion molecules (e.g. ICAM-1, L-selectin) and chemokines (e.g. MIP-1 gamma, CCL5, CCL22) were found (Fig. 2d) that may reflect AT inflammation with recruitment and infiltration of immune cells into the mesenteric WAT.

### KC mice with HSL deficiency have an increased incidence of PDAC

**Fig. 3:**
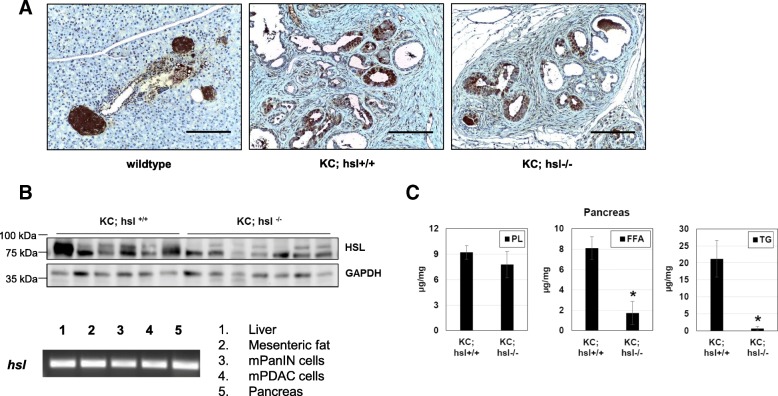
**a** Representative HSL immunohistochemistry of the pancreas in wildtype, *KC;Hsl*^*+/+*^, and *KC;Hsl*^*−/−*^ mice showing HSL positivity in pancreatic islets and PanIN cells. Scale bar represents 100 μm. **b** HSL western blot of total pancreatic lysates in *KC;Hsl*^*+/+*^ and *KC;Hsl*^*−/−*^ mice (GAPDH as loading control). PCR analysis of *Hsl* transcripts in differentiated 3 T3-L1 (adipocytes), murine PanIN (mPanIN) and murine PDAC cells (both isolated from KC mice), murine pancreas and liver (lower). **c** Quantification of PL, TG, and FFA content in the pancreas of *KC;Hsl*^*+/+*^ and *KC;Hsl*^*−/−*^ mice (*n* = 5). *: *p* < 0.01

Consistent with previous reports [21], HSL was expressed in pancreatic islets of wild type (WT) mice (Fig. 3a). Interestingly, immunohistochemistry showed strong HSL expression in PanIN lesions of *KC;Hsl*^*+/+*^ and *KC;Hsl*^*−/−*^ mice (Fig. 3a), which has never been described before. Expression of HSL in PanIN cells was confirmed by PCR analysis of primary PanIN cells isolated from KC mice (with functional HSL) and cultured in vitro (Fig. 3b). Despite the functional HSL deficiency in *KC;Hsl*^*−/−*^ mice (by virtue of replacing portions of exon 5 and the entire exon 6 with a neo cassette), the antibodies used in the present study seem to be able to detect the non-functional HSL protein in *KC;hsl*^*−/−*^ mice. It is unclear whether the apparent lower expression of HSL in *KC;Hsl*^*−/−*^ WAT and pancreatic lysates (Fig. 2c and 3b) is caused by an actual decreased expression or by a lower affinity of the antibody to the non-functional HSL protein. Detailed pancreatic tissue lipid analysis demonstrated markedly reduced TG and FFA content in *KC;Hsl*^*−/−*^ mice (Fig. 3c). Again, the PL content was unaltered in the pancreas of *KC;Hsl*^*−/−*^ mice (Fig. 3c).

**Fig. 4:**
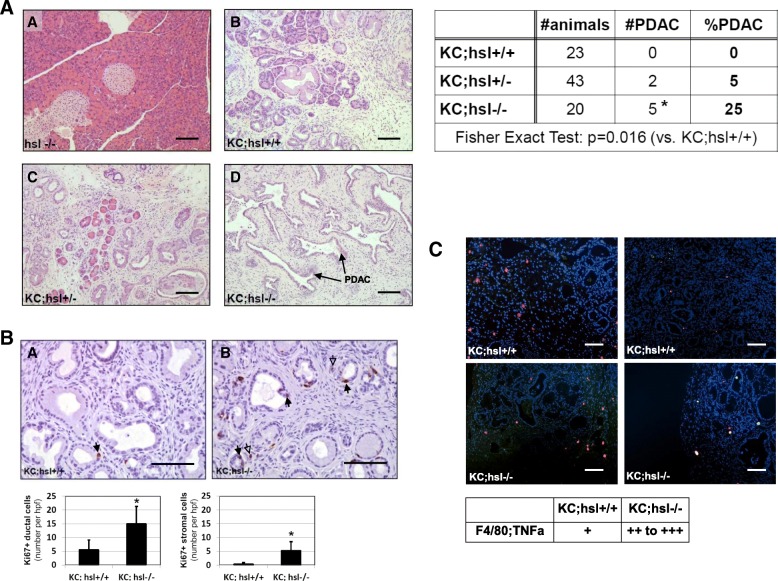
**a** Representative histology (H.E. staining) of the pancreas of *Hsl*^*−/−*^, *KC;Hsl*^*+/+*^, *KC;Hsl*^*+/−*^, and *KC;Hsl*^*−/−*^ mice. Scale bar represents 100 μm. No pancreatic neoplastic lesions were detected in *Hsl*^*−/−*^ mice. Analysis of PDAC incidence at 20 weeks (sacrifice) in *KC;Hsl*^*+/+*^, *KC;Hsl*^*+/−*^, and *KC;Hsl*^*−/−*^ mice (table right). **b** Ki67 immunohistochemistry of the pancreas of *KC;Hsl*^*+/+*^ and *KC;Hsl*^*−/−*^ mice. Scale bar represents 100 μm. Quantification of Ki67 immunoreactivity in epithelial (ductal, indicated by solid arrows) and stromal cells (indicated by hollow arrows). N = 6, *: *p* < 0.01. **c** Immunofluorescence staining of the pancreas of *KC;Hsl*^*+/+*^ and *KC;Hsl*^*−/−*^ mice (two representative mice in each group). Scale bar represents 100 μm. Red and green staining denote F4/80 (macrophage marker) and TNF-α (as a marker of tissue inflammation), respectively. Semi-quantitative analysis of TNF-α expressing macrophages (orange staining) in *KC;Hsl*^*+/+*^ and *KC;Hsl*^*−/−*^ mice (table below)

The most salient feature of this study is that *KC;Hsl*^*−/−*^ mice displayed a significantly increased PDAC incidence at 20 weeks (Fig. 4a). While none of the *KC;Hsl*^*+/+*^ mice developed invasive PDAC at six months of age, 25% (5/20) of *KC;Hsl*^*−/−*^ mice had cancer (*p* = 0.016). The increased PDAC incidence in *KC;Hsl*^*−/−*^ mice was thereby associated with an elevated proliferation of PanIN and stromal cells (Fig. 4b) as assessed by Ki67 immunostaining. Furthermore, *KC;Hsl*^*−/−*^ mice displayed enhanced pancreatic inflammation as evident histologically and by an increase in TNF-α expressing F4/80-positive macrophages assessed by immunofluorescence (Fig. 4c).

### Patients with PDAC and low expression of *LIPE* have unfavorable prognosis

**Fig. 5:**
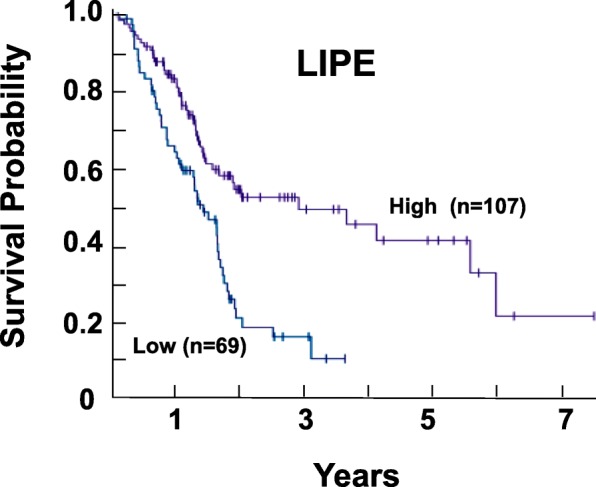
Kaplan-Meier plot for gene expression of *LIPE* in PDAC. Image was reproduced from the Human Protein Atlas (version 17) available from www.proteinatlas.org. The link to *LIPE* is as follows: http://www.proteinatlas.org/ENSG00000079435-LIPE/pathology/tissue/pancreatic+cancer

To assess the significance of HSL expression in human PDAC, we determined the importance of the expression of the gene encoding HSL as a prognostic marker of survival in patients with PDAC. We used a recently published interactive open-access database (www.proteinatlas.org/pathology) to perform correlation analyses based on mRNA expression levels of *LIPE* (the gene encoding HSL) in PDAC tissue and the clinical outcome (survival) of the patients. The data in the Pathology Atlas is based on the analysis of transcriptomics and survival in 176 PDAC patients. As illustrated in the Kaplan-Meier plot in Fig. 5**,** none of the patients of the population with lower levels of *LIPE* mRNA expression (*n* = 69) survived for 5 years while 42% of the population (*n* = 107) with the higher levels of *LIPE* mRNA survived for 5 years or more.

## Discussion

A striking feature of the results presented here is that KC mice lacking HSL (*KC;Hsl*^*−/−*^) mice displayed a significant increase in PDAC incidence. To our knowledge, it is the first time that HSL deficiency has been linked to an increased cancer risk. In humans, carriers of a frameshift deletion of exon 9 in the *LIPE* gene, encoding for HSL, were characterized by metabolic dysfunction, including dyslipidemia, hepatic steatosis, systemic insulin resistance, and diabetes [22]. In the adipose tissue from carriers with the mutation, impaired lipolysis and inflammation were observed [22]. In our study, the exact mechanism(s) involved in promoting PDAC remains incompletely understood. HSL deficiency in KC mice was accompanied by enhanced inflammation in the AT and pancreas. While CLS is a well-characterized feature of adipose tissue inflammation (macrophages surrounding necrotic adipocytes), additional analysis is warranted to further identify inflammatory cell subpopulations. It has been postulated that an increased heterogeneity of adipocytes with necrotic cell death of hypertrophic adipocytes and subsequent infiltration of macrophages may play an important role in inducing WAT inflammation in the context of HSL deficiency [10, 23]. Lipid analysis of the mesenteric WAT in *KC;Hsl*^*−/−*^ mice has revealed a decreased TG content, in agreement with previous reports [24]. The mechanistic link between HSL deficiency and TG reduction in WAT was suggested to be a compensatory downregulation of FA esterification enzymes, leading to reduced cellular TG synthesis [25]. This observation is consistent with the reported impairment of white adipocyte differentiation and decreased WAT mass in HSL null mice [18]. The anti-adipogenic phenotype may also be mediated by a decrease in PPAR-γ expression and activity through the reduced intracellular release of FAs, acting as endogenous PPAR-γ ligands, in HSL null mice [19]. This is corroborated by our finding of reduced PPAR-γ expression in the mesenteric WAT of *KC;Hsl*^*−/−*^ mice. FFA levels in the mesenteric WAT were not significantly altered in *KC;Hsl*^*−/−*^ mice, which can be explained by the presence of additional lipases in the WAT, i.e. adipose triglyceride lipase, which preferentially catalyzes the conversion of triacylglycerol to diacylglycerol and thereby maintaining a FFA pool in WAT [24].

In addition to AT inflammation, *KC;Hsl*^*−/−*^ mice also had enhanced pancreatic inflammation as demonstrated histologically and by an increased number of TNF-α producing macrophages in the pancreas. It is currently unclear what mechanisms elicited the pancreatic inflammation seen in *KC;Hsl*^*−/−*^ mice. It is possible that HSL deficiency causes enhanced inflammation primarily in the visceral WAT adjacent to the pancreas with an increased production and secretion of pro-inflammatory cytokines, which could elicit a subsequent inflammatory reaction in the pancreas of KC mice. This is supported by our finding that HSL deficiency failed to cause pancreatic inflammation in WT mice. It is conceivable that the robust visceral WAT inflammation in *KC;Hsl*^*−/−*^ mice reinforces and amplifies the oncogenic and inflammatory signaling in KrasG12D harboring pancreatic cells, thereby leading to substantial pancreatic inflammation and accelerated PDAC development.

However, we cannot rule out an important role of pancreatic HSL in mediating pancreatic inflammation and tumorigenesis. Indeed, we detected strong HSL expression in pancreatic islets and PanIN lesions and reduced expression of *LIPE* (the gene encoding HSL) in pancreatic tissue of patients with PDAC is associated with decreased overall survival [26]. HSL deficiency in pancreatic islets may thereby explain the observed reduction in insulin of *KC;Hsl*^*−/−*^ mice in our study, as HSL has been described to be important in glucose-stimulated insulin secretion in pancreatic beta cells [27]. HSL deficiency in the pancreas was accompanied by reduced TG and FFA levels, indicating a prominent role of HSL (and a possible absence of additional neutral lipases) in FFA release from intracellular TG depots in the pancreas. It is plausible that in the presence of oncogenic KRAS these lipid changes locally in the pancreas in *KC;Hsl*^*−/−*^ mice are driving promotional factors of pancreatic inflammation and PDAC development. These possibilities are not mutually exclusive and the lack of HSL in both visceral WAT and in the pancreas of KC could cooperate in accelerating PDAC development.

## Conclusion

Our data demonstrated that HSL deficiency in KC mice leads to inflammation of mesenteric WAT and the pancreas and importantly accelerated PDAC formation. Collectively, our results revealed an unexpected tumor-suppressive role of HSL and emphasize the need of caution in targeting HSL for tumor cachexia or dyslipidemia, as chronic suppression of this enzyme may lead to increased incidence of PDAC.

## Abbreviations

PPAR-γ: Peroxisome proliferator-activated receptor gamma
TNF-α: Tumor necrosis factor-alpha
AT: Adipose tissue
CLS: Crown like structures
DIO: Diet-induced obesity
FA: Fatty acid
FFA: Free fatty acid
HPF: High power field
HSL: Hormone sensitive lipase
PanIN: Pancreatic intraepithelial neoplasia
PCR: Polymerase chain reaction
PDAC: Pancreatic ductal adenocarcinoma
PL: Phospholipids
TG: Triglycerides
WAT: White adipose tissue
